# Genomic Prediction of Northern Corn Leaf Blight Resistance in Maize with Combined or Separated Training Sets for Heterotic Groups

**DOI:** 10.1534/g3.112.004630

**Published:** 2013-02-01

**Authors:** Frank Technow, Anna Bürger, Albrecht E. Melchinger

**Affiliations:** Institute of Plant Breeding, Seed Sciences and Population Genetics, University of Hohenheim, Stuttgart 70599, Germany

**Keywords:** genomic prediction, maize, disease resistance, northern corn leaf blight, heterotic groups, GenPred, shared data resources

## Abstract

Northern corn leaf blight (NCLB), a severe fungal disease causing yield losses worldwide, is most effectively controlled by resistant varieties. Genomic prediction could greatly aid resistance breeding efforts. However, the development of accurate prediction models requires large training sets of genotyped and phenotyped individuals. Maize hybrid breeding is based on distinct heterotic groups that maximize heterosis (the dent and flint groups in Central Europe). The resulting allocation of resources to parallel breeding programs challenges the establishment of sufficiently sized training sets within groups. Therefore, using training sets combining both heterotic groups might be a possibility of increasing training set sizes and thereby prediction accuracies. The objectives of our study were to assess the prospect of genomic prediction of NCLB resistance in maize and the benefit of a training set that combines two heterotic groups. Our data comprised 100 dent and 97 flint lines, phenotyped for NCLB resistance *per se* and genotyped with high-density single-nucleotide polymorphism marker data. A genomic BLUP model was used to predict genotypic values. Prediction accuracies reached a maximum of 0.706 (dent) and 0.690 (flint), and there was a strong positive response to increases in training set size. The use of combined training sets led to significantly greater prediction accuracies for both heterotic groups. Our results encourage the application of genomic prediction in NCLB-resistance breeding programs and the use of combined training sets.

Northern corn leaf blight (NCLB), caused by the pathogen *Setosphaeria turcica* (anamorph *Exserohilum turcicum*), is a serious threat to maize (*Zea mays* L.) cultivation worldwide, reportedly causing yield losses of more than 50% (Raymundo and Hooker 1981; Perkins and Pederson 1987). NCLB can be efficiently controlled through cultivation of resistant varieties (Dingerdissen *et al.* 1996), giving breeding for NCLB resistance a high priority for disease control. Today’s availability of high-density molecular marker data greatly facilitates molecular resistance breeding approaches (Collard and Mackill 2008) and the understanding of the genetic architecture of resistance traits. Previous studies on resistance to NCLB point to a complex genetic architecture with many quantitative trait loci (QTL) distributed throughout the genome (Van Inghelandt *et al.* 2012; Poland *et al.* 2011; Wisser *et al.* 2006). For instance, Poland *et al.* (2011) identified 29 QTL for NCLB resistance, each with a small effect. This might hamper the application of traditional marker assisted breeding approaches.

Genomic prediction, developed in dairy cattle breeding, uses all available marker data of a genotyped and phenotyped training set for building a prediction model without an intermediate QTL detection step (Meuwissen *et al.* 2001). Subsequently, this model is used to predict genotypic values of nonphenotyped individuals for which only marker data are available. The major advantage of genomic prediction is that all polymorphisms affecting a trait are modeled, regardless of effect size, making it a potentially powerful approach for a complex trait like NCLB resistance.

Initial studies on genomic prediction applied to maize showed promising results with highly accurate predictions for traits like dry matter yield and plant height (Riedelsheimer *et al.* 2012; Albrecht *et al.* 2011). Although no results are available for genomic prediction of disease resistance in maize, it has been successfully applied to predict resistance to *Fusarium* head blight (FHB) in barley (Lorenz *et al.* 2012) and wheat (Rutkoski *et al.* 2012).

In dairy cattle breeding, genomic prediction is now applied routinely for large breeding populations like Holstein Friesian (Hayes *et al.* 2009b). However, its application to small breeds seems to be more challenging, mainly because difficulties of assembling large-enough training sets. To make the advantages of genomic prediction available for small breeds as well, the possibility of combined, multibreed training set were studied by several authors (Erbe *et al.* 2012; Weber *et al.* 2012; de Roos *et al.* 2009; Hayes *et al.* 2009a). These authors found this approach to have the potential of increasing the prediction accuracies for small breeds. The approach of using a training set that combines different groups also has been studied in a plant breeding context for genomic prediction of oat (Asoro *et al.* 2011) and barley (Lorenz *et al.* 2012). The results of these studies, however, were inconclusive.

In maize breeding, the two parental lines of a hybrid are taken from genetically distinct heterotic groups (dent and flint in Central Europe) for maximum exploitation of heterosis (Messmer *et al.* 1993). For resistance traits with mainly additive gene action, as applies to NCLB resistance (Carson 1995), both parents of a hybrid should have good resistance. The improvement of the resistance level of the inbred lines within each heterotic group requires allocating the available resources to parallel breeding programs. This makes it more challenging to establish a sufficiently sized training set within each heterotic group. Therefore, enlarging the training set via combination of data from both heterotic groups also would be an interesting approach for genomic prediction in maize breeding. The objectives of this study were to (1) assess the prospects of genomic prediction of NCLB resistance in maize and (2) compare the prediction accuracy of separate training sets for each heterotic group *vs.* combining both heterotic groups in a single training set.

## Materials and Methods

### Plant material and phenotypic evaluation

Our genetic material consisted of 100 dent and 97 flint maize inbred lines, representing the breeding program of the University of Hohenheim. More detailed information on the history of this breeding program is given by Technow *et al.* (2012). All lines were evaluated for NCLB resistance *per se* in the trial stations Bingen (Rhineland-Palatinate, Germany) and Pocking (Bavaria, Germany) in 2010. Plants were grown in single row plots, laid out in a 20 × 10 alpha-design with two replications at each location. *E. turcicum* inoculum was artificially applied using pathogen extract of naturally infected leafs collected at each location in 2009. NCLB severity was visually rated for each plot according to the lesion spot development in the middle-to-upper part leaves on a scale from 1 (susceptible) to 9 (resistant). NCLB severity ratings were adjusted for effects pertaining to the environment and field design. The dent heterotic group had a phenotypic mean of 3.28 (range, 0.98−6.45), the flint heterotic group had a phenotypic mean of 3.77 (range, 1.29−5.84). The heritability (*H*^2^) on an entry mean basis was 0.76 for dent and 0.64 for flint. The adjusted entry means, computed as best linear unbiased estimates by using a mixed model with genotypes treated as fixed effects, are provided in Supporting Information, File S1.

### Genomic data

All inbred lines were genotyped with the Illumina single-nucleotide polymorphism (SNP) chip MaizeSNP50 (Ganal *et al.* 2011) containing 57,841 SNPs. Markers with more than 5% missing data within any heterotic group were removed. Because the inbred lines were in very advanced selfing generations (> S_6_), heterozygous marker genotypes also were treated as genotyping errors and considered as missing. “BEAGLE” software (Browning and Browning 2009), version 3.3.1, was used to impute all remaining missing marker genotypes, resulting in 37,908 SNP markers available for further analysis.

Second-order natural smoothing spline regression was used to visualize the linkage disequilibrium (LD, calculated as *r*^2^) as a function of the physical distance Δ in Mbp between markers on the same chromosome. This was done separately for the set of dent lines, flint lines, and across both sets. For LD calculations within heterotic groups, all markers with a minor allele frequency (MAF) > 0.05 within the corresponding group and for LD calculation across groups all markers with a MAF > 0.05 within both heterotic groups were included.

The linkage phase persistence across heterotic groups was computed following Technow *et al.* (2012). First, all marker pairs were binned according to the physical distance Δ in 100 discrete bins of 0.05 Mbp width. Second, the proportion of marker pairs, with identical linkage phase within both heterotic groups, was calculated for each bin. Again, second-order natural smoothing spline regression was used to visualize this proportion as a function of the distance between the center values of the bins.

A principal component analysis, based on the full 37,908 SNP marker profiles of the inbred lines, was used to investigate the genetic distinction of the dent and flint heterotic groups.

### Prediction approaches and their validation

**Figure 1:**
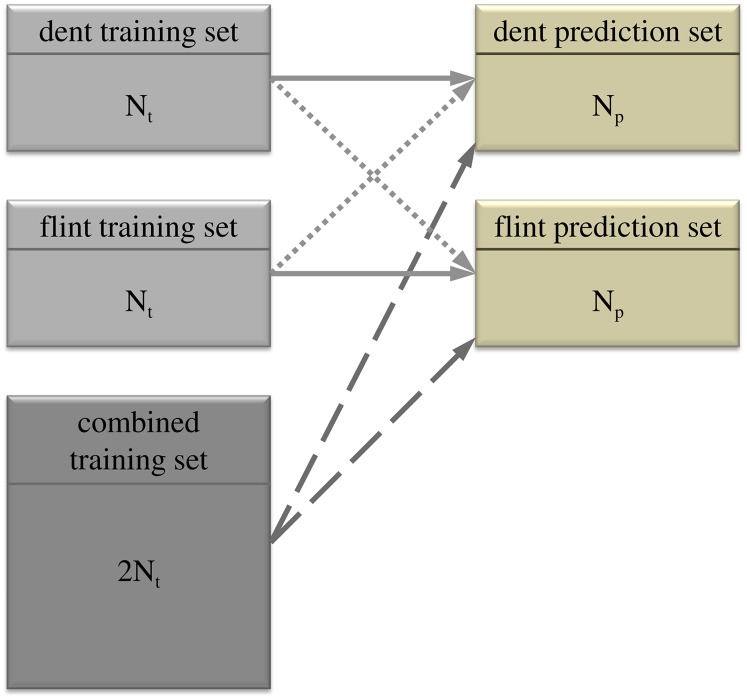
Schematic illustration of the investigated prediction approaches: “within” prediction approach (full line), “across” prediction approach (dotted line) and “combined” prediction approach (dashed line). *N_t_* corresponds to the training set size and *N_p_* to the size of the prediction set.

We investigated the potential of the following three prediction approaches (Figure 1): (1) the “within” prediction approach, where lines used for fitting the model (training set) and lines to be predicted (prediction set) belonged to the same heterotic group; (2) the “across” prediction approach, where lines in the training set belonged to another heterotic group than lines in the predicting set and; (3) the “combined” prediction approach, where lines of both heterotic groups were combined in a training set to predict either lines in a flint or dent prediction set. The dent and flint training sets comprised a random sample of *N_t_* = 75 (25, 50) lines from the corresponding group. The remaining dent and flint lines then made up the prediction sets. The training sets for the “combined” prediction approach were created by merging the training sets from both heterotic groups (Figure 1). The “prediction accuracy” was calculated by dividing the correlation between the predicted genotypic values and observed phenotypic values by $\sqrt{H^{2}}$, following common practice (Legarra *et al.* 2008). The process of generating training and prediction sets was repeated 100 times for all three levels of *N_t_* in the manner described. All prediction approaches were applied to the same random splits of the data set into training and prediction set, and a paired *t*-test was used to determine the significance of differences in prediction accuracy observed between the “combined” and “within” prediction approaches. Because the training and prediction sets produced over the replications are always drawn from the same data set, the replications are not independent. To account for this, we used the correction method proposed by Nadeau and Bengio (2003). Their adjustment is the stronger, the larger the size of the prediction set (*N_p_*) is relative to *N_t_*, because the larger *N_p_*/*N_t_*, the more overlapping the sampled prediction sets will be. Therefore, the test is very conservative for *N_t_* = 25 and *N_t_* = 50.

### Prediction model

The Bayesian version of genomic best linear unbiased prediction (Kärkkäinen and Sillanpää 2012) was used to predicted genotypic values as

$$\mu_{i}=X_{i}\beta +u_{i}$$

(1) $$y_{i}∼N\left(\mu_{i},\sigma_{e}^{2}\right),$$

where *μ_i_* denotes the linear predictor, *y_i_* is the scaled and centered phenotypic entry mean of inbred line *i* and *u_i_* its total genetic value. The Gaussian density function is denoted by N and $\sigma_{e}^{2}$ refers to the residual variance. The design row vector ***X****_i_* codes for the fixed effects in ***β***. Depending on the prediction approach, these were either the heterotic group effects (“combined” prediction approach) or a single intercept (“within” and ’across’ prediction approaches). We used a uniform, improper prior for ***β***. The prior for *u_i_* was $ℳVN\left(0,A\sigma_{u}^{2}\right)$, where ℳVN denotes the multivariate-Gaussian density function, $\sigma_{u}^{2}$ the polygenic variance, and ***A*** the realized additive relationship matrix. The latter was computed from the marker data according to Method 1 of VanRaden (2008). Finally, the priors for the variance components $\sigma_{e}^{2}$ and $\sigma_{u}^{2}$ were uninformative scaled inverse χ^2^ distributions with scale factor equal to 1/2 and degree of freedom parameter equal to 2.

A single Gibbs-sampling chain run for 50,000 iterations was used for sampling from the marginal posterior distributions of the parameters involved. The first 20,000 iterations of the chain were discarded as burn-in, and only every 20^th^ sample of the remaining iterations was stored. The posterior means of ***β*** and *u_i_* were used to predict the genotypic values. The R (R Development Core Team 2011) package “MCMCglmm” (Hadfield 2010) was used for Gibbs-sampling.

### Computation of realized additive relationship matrix

Only markers informative for a given prediction approach were considered for computing ***A***. Consequently, markers had to segregate (always meaning MAF > 0.05) in at least one heterotic group for the “combined” prediction approach, in both heterotic groups for the “across” prediction approach and in the corresponding heterotic group for the “within” prediction approach. Because the markers were distributed unevenly across the genome, the number of markers was reduced to a density of one marker per Mbp, with a distance of approximately 1 Mbp between adjacent markers, to ensure equal weighing of all genomic regions when computing ***A***. This resulted in a total of 1724 (“combined”), 1513 (“across”), and 1638 (“within”) markers finally used. These marker data sets are provided in File S1.

## Results

### LD and genetic relationship between lines

**Figure 2:**
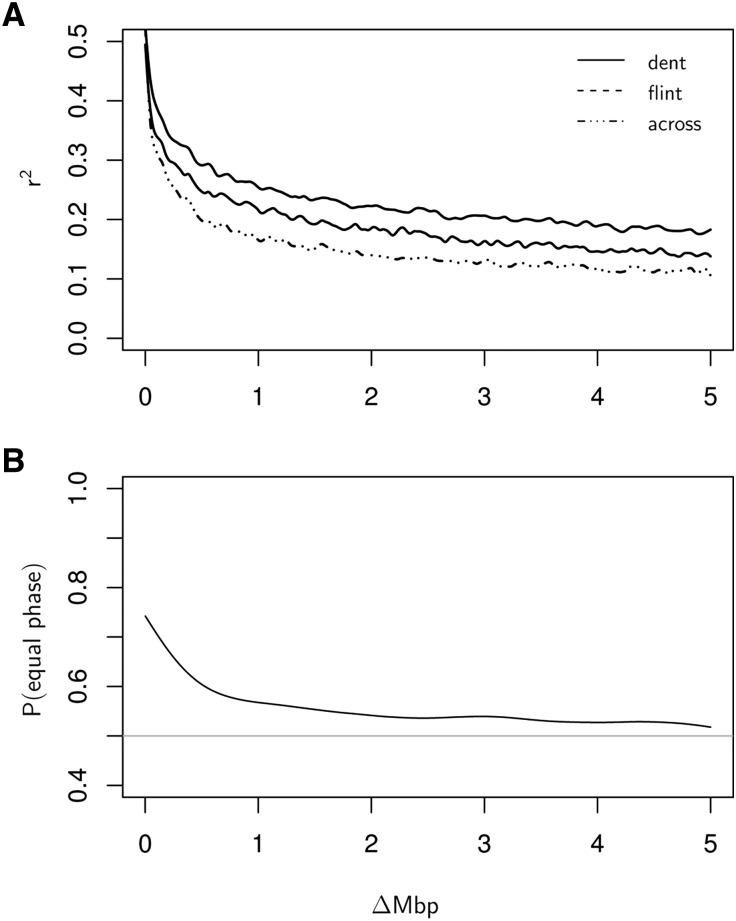
(A) LD (calculated as *r*^2^) as a function of physical distance (Δ) in Mbp between markers on the same chromosome for the group of dent lines (full line), flint lines (dashed line), and across both heterotic groups (dotted-dashed line). (B) Proportion of markers with equal linkage phase across heterotic groups as a function of Δ in Mbp between markers on the same chromosome. The horizontal gray line indicates the value 0.5. LD calculations within heterotic groups included all markers with MAF > 0.05 within this group; LD calculation across groups included all markers with MAF > 0.05 within both heterotic groups.

LD between markers with Δ less than 0.5 Mbp was at very high levels of greater than 0.30 (Figure 2A). It then decreased but still amounted to ≈0.25 within heterotic groups and ≈0.20 across at Δ = 1.0 Mbp. Beyond Δ = 1.0 Mbp, LD continued to decrease slightly but remained considerably greater than a value of 0.10 for the whole range of Δ values considered. In general, the LD within the group of dent lines was slightly greater compared with the group of flint lines, whereas the LD across the set of dent and flint lines was lowest (Figure 2A).

The proportion of marker pairs with the same linkage phase in both heterotic groups showed trends similar to the LD (Figure 2B). It reached a maximum of ≈ 0.75 for marker pairs with a distance Δ in Mbp close to zero and then decreased rather quickly. However, at Δ = 1.0 Mbp it still remained just below 0.6. Afterward, it decreased slowly toward the value 0.5 but nonetheless remained slightly above this value over the whole range of Δ values considered.

**Figure 3:**
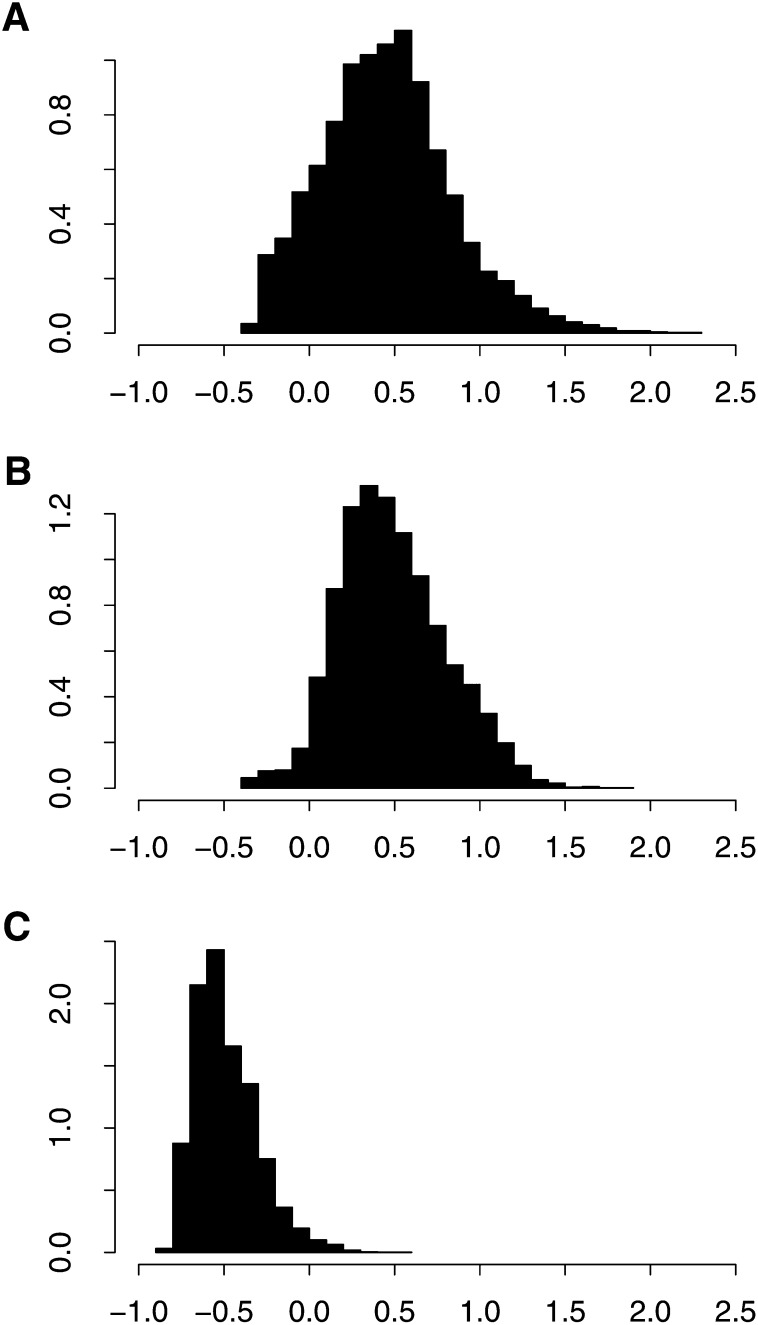
Density histograms of pairwise relationship coefficients between dent lines (A), flint lines (B) and between dent and flint lines (C). Values are elements of the realized additive relationship matrix ***A*** as computed for the “combined” prediction approach.

**Figure 4:**
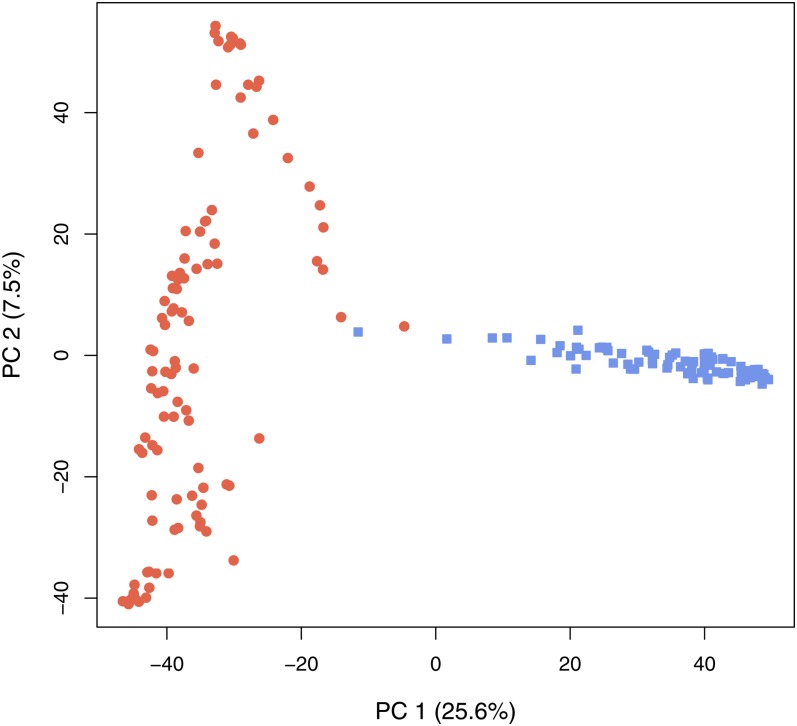
Plot of principal component (PC) 1 *vs.* PC 2 scores based on 37,908 SNP markers of all 100 dent lines (red dots) and 97 flint lines (blue squares).

The mean pairwise relationship coefficient, from the ***A*** matrix computed for the “combined” prediction approach, between dent lines was 0.46 with standard deviation of 0.38, and between flint lines 0.49 with standard deviation of 0.32. Between the dent and flint lines, the mean was −0.49 with standard deviation of 0.18 (Figure 3). The principal component analysis showed a very clear genetic distinction of the dent and flint heterotic groups (Figure 4).

### Prediction accuracy

Overall, prediction accuracies increased with increasing *N_t_*. For example, the prediction accuracy of dent (flint) lines increased from 0.366 (0.389) at *N_t_* = 25 to 0.589 (0.576) at *N_t_* = 50 to 0.706 (0.690) at *N_t_* = 75 (Table 1, combined training sets). The “combined” prediction approach resulted in greater prediction accuracies than those of the “within” prediction approach for all levels of *N_t_*. Thereby, the largest differences were observed at *N_t_* = 75 with 0.065 for dent lines and 0.082 for flint lines (Table 1). These differences were also statistically significant (*P* < 0.05).

**Table 1:** Average and SD of prediction accuracies over the 100 replications of the validation procedure for northern corn leaf blight resistance based on a Bayesian GBLUP model using either pure dent, pure flint, or combined training sets of size *N_t_* to predict either the dent or flint lines

Training Set	Prediction Set	*N_t_* = 25	*N_t_* = 50	*N_t_* = 75
Dent (*N_t_*)	Dent	0.325*^a^* ± 0.125	0.532*^a^* ± 0.112	0.641*^a^* ± 0.131
	Flint	0.084 ± 0.205	0.210 ± 0.213	0.292 ± 0.257
Flint (*N_t_*)	Dent	0.093 ± 0.110	0.078 ± 0.150	0.110 ± 0.279
	Flint	0.340^*A*^ ± 0.151	0.498*^A^* ± 0.133	0.608*^A^* ± 0.156
Combined (2*N_t_*)	Dent	0.366*^a^* ± 0.123	0.589*^a^* ± 0.097	0.706*^b^* ± 0.114
	Flint	0.389^*A*^ ± 0.144	0.576*^A^* ± 0.117	0.690*^B^* ± 0.157

Values followed by identical letters within a column are not statistically different in adjusted paired *t*-tests for *P* < 0.05. The comparisons considered were (1) within and combined prediction approach for dent (lowercase superscript letters) and (2) within and combined prediction approach for flint (uppercase superscript letters). GBLUP, genomic best linear unbiased prediction.

Prediction accuracies of the “across” prediction approach were relatively low in both cases, whereby prediction of flint lines using a dent training set was more accurate than vice versa.

## Discussion

### Merit of selection based on genomic prediction

Successful adoption of genomic prediction approaches to plant breeding programs depends on their advantage over traditional selection methods. For quantifying this potential advantage, genomic prediction can be viewed as an indirect selection method. The merit of indirect selection per unit time, relative to the merit of direct selection, can be described as the indirect selection response (*CR_X_*) divided by the direct selection response (*R_X_*). Following Falconer and Mackay (1996), this ratio can be calculated as

(2) $$\frac{CR_{X}}{R_{X}}=\frac{i_{Y}\;H_{Y}\;r_{A}\;L_{X}}{i_{X}\;H_{X}\;L_{Y}}$$

where *i_Y_* is the selection intensity applied on the indirect trait and *i_X_* the selection intensity on the target trait, *L_Y_* and *L_X_* are the cycle lengths of indirect and direct selection, respectively, *H_X_* is the square root of the heritability of the target trait, and *H_Y_* the square root of the heritability of the indirect trait. The latter is assumed to be 1 in the case of genomic data. The genetic correlation between the target and indirect trait is denoted by *r_A_* and corresponds to the prediction accuracy in our context. A ratio greater than 1 indicates superiority of indirect selection over direct selection. Assuming equal selection intensities, we can arrange equation (2) to the inequality

(3) $$L_{Y}<\frac{r_{A}}{H_{X}}L_{X}.$$

It describes the merit of indirect selection as a function of the selection cycle lengths. Accordingly, indirect selection is superior to direct selection when *L_Y_* is shorter than a certain fraction of *L_X_*, which depends on *H*^2^ of the target trait and the accuracy of genomic predictions.

Using our *H*^2^ estimates and the accuracies observed for the “combined” prediction approach at *N_t_* = 75, selection for NCLB resistance based on genomic predictions would already be superior to phenotypic selection when *L_Y_* is less than 81% (dent) or 86% (flint) of *L_X_*. These are promising numbers, given that other authors found genome based breeding programs to require less than 50% of the time as traditional programs (Heffner *et al.* 2010).

Equation (3) assumed that *i_Y_* = *i_X_*. However, after sufficiently sized training sets are established, which requires phenotypic as well as genotypic data, *i_Y_* can be raised almost arbitrarily by genotyping large numbers of individuals. When novel techniques such as genotyping-by-sequencing are used, this could be done at very competitive costs (Elshire *et al.* 2011). Thus, in the near future, it is likely that *i_Y_* > *i_X_*, which would add to the advantage of selection based on genomic predictions.

The potential advantage of selection based on genomic predictions was also pointed out by other authors. For example, in simulation studies, Heffner *et al.* (2010) and Bernardo and Yu (2007) found genomic breeding programs to be clearly superior over traditional recurrent selection programs for complex traits in maize.

Our results and conclusions are based on rather low training set sizes *N_t_*. Other studies in crop species too reported high prediction accuracies with surprisingly low *N_t_* (Zhao *et al.* 2011; Lorenz *et al.* 2012). One likely explanation for this are the commonly low effective population sizes *N_e_* and consequently low effective number of independent chromosome segments *M_e_*, found in plant breeding populations (Riedelsheimer *et al.* 2012). Following Daetwyler *et al.* (2010b), the expected prediction accuracy can be calculated as

(4) $$\sqrt{\frac{N_{t}H^{2}}{N_{t}H^{2}+M_{e}}}$$

where *M_e_* = 2*N_e_G*/*log*(4*N_e_G*) and *G* is the genome length in Morgan. From equation (4), it can be seen that low *M_e_* combined with high *H*^2^ can lead to a high expected prediction accuracy at low *N_t_*. For example, with *N_e_* = 25, which is at the upper end of the range postulated in populations of maize inbred lines (Guzman and Lamkey 2000), and a genome length of 16.34 Morgan (Martin *et al.* 2011), the expected prediction accuracy at *N_t_* = 25 is 0.38 (dent) and 0.35 (flint) and 0.58 (dent) and 0.55 (flint) at *N_t_* = 75. These values agree well with our results. Nevertheless, *N_t_* will likely be greater in routine applications by plant breeders. This is expected to increase the prediction accuracy, and thereby the merit of selection based on genomic predictions, even further.

### Merit of combined training sets

In accordance with other studies on genomic prediction in crops (Asoro *et al.* 2011) and livestock (Weber *et al.* 2012; Erbe *et al.* 2012; Daetwyler *et al.* 2010a; de Roos *et al.* 2009; Hayes *et al.* 2009a), we observed an increase in prediction accuracy when using a combined training set as compared with using training sets comprising lines from one heterotic group only.

Interestingly, this was already the case at a comparatively low marker density of 1 Mbp^–1^ or about 1600–1700 markers. However, there is consensus among the aforementioned authors that very high marker densities are required to take advantage of combined training sets. This is to ensure consistent linkage phases between QTL and markers across groups, a necessary condition for the combined prediction approach to work. However, despite several centuries of separation of dent and flint (Rebourg *et al.* 2003), our results showed that there is still consistent LD across the heterotic groups, even for markers at greater distances (*i.e.*, the proportion of marker pairs with equal linkage phase was considerably greater than 0.5, the value representing independence). Further, we did not find that using higher marker densities led to an increase in the prediction accuracy (results not shown). Similar results on the required marker density for prediction purposes in elite germplasm of maize were reported by Riedelsheimer *et al.* (2012), who found that the prediction accuracy did not increase markedly when increasing the number of markers beyond 2500.

Genomic best linear unbiased prediction, the prediction method used by us, uses marker data merely for estimating the realized relationship between individuals. Marker effects based methods (Kärkkäinen and Sillanpää 2012) might be able to capitalize more on higher marker densities (Erbe *et al.* 2012). However, using a ‘BayesB’ type algorithm (Meuwissen *et al.* 2001) in the implementation employed by Technow *et al.* (2012) did not yield greater prediction accuracies (results not shown). Likely, this was because such algorithms require much larger training set sizes to overcome the added complexity of the model due to greater dimensionality and redundancy of the predictor set.

Lorenz *et al.* (2012) studied the potential of combined training sets for predicting resistance to FHB and related deoxynivalenol toxin (DON) production in barley. They did not find that using a combined training set of 200 individuals from two groups increased the prediction accuracy over using just 100 individuals from either group for predicting individuals from the same group. However, their populations seemed to be rather unresponsive to increases in training set size *N_t_*. For example, even doubling *N_t_* from 100 to 200 for prediction within a group just barely increased the prediction accuracy (4% for FHB and 10% for DON). Therefore, combining 100 + 100 individuals from different groups should not be expected to yield much improvement either. Focusing on prediction accuracies within heterotic groups, we found that increasing *N_t_* from 25 to 50 increased prediction accuracies by 64% (dent) and 46% (flint). In contrast, the same increase in *N_t_* for the populations of Lorenz *et al.* (2012), increased their prediction accuracies just by 17% and 26% for FHB and DON respectively. Further, we observed a relative increase in accuracy from *N_t_* = 50 to *N_t_* = 75 that was considerably greater than the relative increase Lorenz *et al.* (2012) observed from *N_t_* = 50 to *N_t_* = 100. Thus, responsiveness to *N_t_*, which may be a function of the effective population size, seems to be a key requirement for an advantage of combined training sets.

Compared with the increase in prediction accuracy when increasing *N_t_* by adding individuals from the same group (*e.g.*, moving from *N_t_* = 25 to *N_t_* = 50 within groups), the increase in prediction accuracy was only marginal, when the same increase in *N_t_* was achieved by adding individuals from the other group. This was because the information added in the latter case was much lower than in the former, as is exemplified in the low linkage phase consistency between the heterotic groups. It is reasonable to assume, however, that the increase would have been more pronounced when the groups were less genetically distant then our dent and flint groups.

Nonetheless, under a fixed budget that has to be allocated to all heterotic groups, increasing *N_t_* within one group can only be achieved by decreasing it in another. This would necessarily lead to differential selection progress, which is undesirable when the heterotic groups are developed reciprocally. Therefore, combining training sets is still worthwhile, since the gain in prediction accuracy obtained is essentially cost neutral and does not lead to a negligence of the other group.

Balancing the large increase in prediction accuracy when moving from *N_t_* to 2*N_t_* within one group and the goal of even development of both groups; however, is possible with an alternating selection scheme. Here, the full phenotyping capacity would be applied to one group in one cycle and to the other group in the next cycle and so on. Thus, always one group would be selected based on a training set of size 2*N_t_* from the same group and one based on across group predictions. Following Falconer and Mackay (1996) and assuming constant selection intensities, heritabilities and genetic variances across cycles, the aforementioned alternating scheme would lead to a greater selection gain over two cycles than a scheme based on the combined prediction approach, when $r_{w,2N_{t}}+r_{a,2N_{t}}>2r_{c,N_{t}}$ ($r_{w,2N_{t}}$ denotes the within group prediction accuracy at 2*N_t_*, $r_{a,2N_{t}}$the across group prediction accuracy when the training set size in the other group is 2*N_t_* and $r_{c,N_{t}}$ the prediction accuracy from the combined training sets, when each group has a training set size of *N_t_*). From our results for *N_t_* = 25 at least, however, the alternating scheme would be inferior for both groups. For the alternating scheme to succeed, the across group prediction accuracy needs to be greater than observed by us. As is the case for the gain from combined training sets, the across group prediction accuracy will likely be the greater the lower the genetic distance between groups is.

In conclusion, our results encourage the application of genomic prediction in a NCLB resistance breeding program. Furthermore, combining maize heterotic groups into a single training set is a promising approach for increasing the prediction accuracy of NCLB resistance.

## Supplementary Material

Supporting Information
