# Supplementary material for: Genomic Prediction of Northern Corn Leaf Blight Resistance in Maize with Combined or Separated Training Sets for Heterotic Groups
Source: G3 (Bethesda). 2013 Feb 1;3(2):197–203. doi: 10.1534/g3.112.004630 (PMC3564980; doi:10.1534/g3.112.004630)
Supplement: Supporting Information [file supp_3_2_197__index.html]

Supporting Information 

# Genomic Prediction of Northern Corn Leaf Blight Resistance in Maize with Combined or Separated Training Sets for Heterotic Groups

## Supporting Information for Technow, Burger, and Melchinger, 2013

**Files in this Data Supplement:**

- File S1 - Supporting data (.zip, 189 KB)
